# DZ2002 ameliorates fibrosis, inflammation, and vasculopathy in experimental systemic sclerosis models

**DOI:** 10.1186/s13075-019-2074-9

**Published:** 2019-12-16

**Authors:** Zongwang Zhang, Yanwei Wu, Bing Wu, Qing Qi, Heng Li, Huimin Lu, Chen Fan, Chunlan Feng, Jianping Zuo, Lili Niu, Wei Tang

**Affiliations:** 1 School of Life Sciences, Shanghai University, 333 Nanchen Road, Baoshan District, Shanghai, 200444 China; 2 Laboratory of Immunopharmacology, Shanghai Institute of Materia Medica, Chinese Academy of Sciences, Shanghai, 201203 China; 3 School of Pharmacy, University of Chinese Academy of Sciences, Beijing, 100049 China

**Keywords:** Systemic sclerosis, SAHH inhibitor, Fibrosis, Inflammation, Vasculopathy, TGF-β

## Abstract

**Background:**

Systemic sclerosis is a multisystem inflammatory and vascular lesion leading to extensive tissue fibrosis. A reversible S-adenosyl-l-homocysteine hydrolase (SAHH) inhibitor, DZ2002, modulates the pathologic processes of various inflammatory diseases and autoimmune diseases. This study is designed to investigate the therapeutic potentiality of DZ2002 for experimental systemic sclerosis models.

**Methods:**

The anti-inflammatory and anti-fibrotic features of DZ2002 and its mechanisms were investigated in a bleomycin (BLM)-induced dermal fibrosis mice model. The effects of DZ2002 on expression of extracellular matrix components and TGF-β signaling in human dermal fibroblasts were analyzed. Simultaneously, the effects of DZ2002 on macrophage activation and endothelial cell adhesion molecule expression were also evaluated.

**Results:**

DZ2002 significantly attenuated dermal fibrosis in BLM-induced mice. Consistently, DZ2002 inhibited the expression of various molecules associated with dermal fibrosis, including transforming growth factor β1, connective tissue growth factor, tumor necrosis factor-α, interferon-γ, IL-1β, IL-4, IL-6, IL-10, IL-12p40, IL-17A, and monocyte chemotactic protein 1 in the lesional skin of BLM-induced mice. Furthermore, DZ2002 decreased the proportion of macrophages, neutrophils, and T cells (especially T helper cells) in the skin tissue of BLM-induced mice. In addition, DZ2002 attenuated both M1 macrophage and M2 macrophage differentiation in vivo and in vitro. Importantly, DZ2002 directly reversed the profibrotic phenotype of transforming growth factor-β1-treated dermal fibroblasts and suppressed ICAM-1, VCAM-1, VEGF, bFGF, and ET-1 expression in endothelial cells. Finally, our investigations showed that DZ2002 relieved systemic sclerosis by regulating fibrosis TGF-β/Smad signaling pathway.

**Conclusions:**

DZ2002 prevents the development of experimental dermal fibrosis by reversing the profibrotic phenotype of various cell types and would be a potential drug for the treatment of systemic sclerosis.

## Background

Systemic sclerosis (SSc), also known as scleroderma, is a systemic autoimmune disease characterized by early inflammation and vascular injury followed by fibrosis of the skin and visceral organs [1–3]. Early SSc is dominated by immune cell activation, followed by secreting pro-inflammatory cytokines that activate endothelial cells and causes upregulation of adhesion molecules. The adhesion molecules in turn recruit immune cells. Continuously activated immune cells secrete profibrotic cytokines to activate fibroblasts and transform myofibroblast, which are caused by a complex series of signal pathways initiated by a number of profibrotic molecules, including transforming growth factor-β (TGF-β), connective tissue growth factor (CTGF), endothelin-1 (ET-1), interleukin (IL)-4, IL-6, and IL-13 [4, 5]. Currently, the scant treatment method is a serious challenge for SSc.

Macrophages and T cells (especially T helper cells) are involved in the process of SSc [6–8]. Monocyte and macrophage phenotypes are highly heterogeneous and can be dynamically regulated by the microenvironment in SSc [9, 10]. Monocytes can differentiate into macrophages in response to various stimuli dependent on the tissue microenvironment after infiltrating the tissue. The differentiated macrophages can be classified as classically activated inflammatory macrophages (M1) and alternatively activated tissue profibrotic macrophages (M2) [11]. M1 macrophages can promote tissue inflammation by producing pro-inflammatory cytokines, including tumor necrosis factor (TNF)-α, IL-6, and IL-12. M2 macrophages can induce or maintain tissue fibrosis by producing profibrotic cytokines, including TGF-β, IL-4, and IL-13. T helper (Th) 1 cells and their secreted interferon-γ (IFN-γ) are effective anti-fibrosis factors [12]. Th17 cells promote inflammation by secreting IL-17A, IL-21, and IL-22, resulting in the development and progression of SSc [13]. In reverse, in SSc, Th2 cells promote the polarization of M2 by producing IL-4 and IL-13, while M2 macrophages produce IL-13 to promote Th2 differentiation, creating a positive feedback loop [14]. Tregs can produce TGF-β1 and IL-10, which are essential for maintaining self-tolerance and preventing autoimmunity [15]. Overall, regulating Th1/Th2/Th17 cell balance and inhibiting macrophage polarization are effective strategies for the treatment of SSc.

It is reported that intercellular adhesion molecule 1 (ICAM-1) and vascular cell adhesion molecule 1 (VCAM-1) elevated in SSc [16–18]. It has been demonstrated that ICAM-1 expression by fibroblasts and endothelial cells were induced by a number of the cytokines important in SSc pathogenesis (TNF-α, IFN-γ, IL-1β, and IL-17). Similarly, VCAM-1 expression can be induced by TNF-α in a dose-dependent manner [19]. Once induced, ICAM-1 and VCAM-1 subsequently recruit and activate monocytes to repair damaged endothelial cells [20, 21]. Monocytes can then boost the co-stimulation and transmigration of inflammatory cells into the extracellular matrix (ECM) and contribute to dysregulated angiogenesis [20, 21]. When overexpressed, these adhesion molecules can be detected in a circulating soluble form and are considered markers of underlying endothelial activity and damage. Hence, ICAM-1 and VCAM-1 expression are associated with SSc disease activity and severity.

SAHH and its substrate S-adenosyl-l-homocysteine (SAH) are deeply involved in the process of transmethylation mediated by S-adenosylmethionine (SAM). Immune cells are especially prone to methylation [22], and modifications of DNA and protein by methylation are key factors in immune responses in the progression of inflammatory disease [23, 24]. Till now, three types of SAHH inhibitors have been described: the irreversible type I, type II inhibitors, and the reversible type III inhibitors. Because of the relatively long turnover rate of SAHH, the irreversible inhibitors manifest significant toxicity, whereas toxicity of type III inhibitors show greatly lowered yet still retain a parallel ability to block the enzyme’s activity [25]. The reversible type III SAHH inhibitor DZ2002 [methyl-(adenin-9-yl)-2-hydroxybutanoate] has been found to have an immunomodulatory function and to alleviate disease in several inflammatory and autoimmune animal models [25–29]. It is reported that DZ2002 prevented lupus-like disease from developing in both BXSB and MRL-Faslpr mouse models and DZ2002 ameliorated imiquimod-induced psoriasis-like skin lesions in mice via suppression of T cell-derived IL-17 [24, 30]. These hinted that methylation inhibition might be an approach for the treatment of autoimmune diseases.

Given that DZ2002 presents a prominent activity of anti-inflammatory and immunosuppression, DZ2002 may have potential therapeutic effects on SSc. To assess this hypothesis, we here investigated the effects of DZ2002 on dermal fibrosis of a BLM-induced mice model of SSc by focusing on the three major pathologic characteristics of SSc: fibrosis, immune abnormalities, and vasculopathy.

## Methods

### Mice

Wild-type C57BL/6 mice were purchased from SLRC Laboratory (Shanghai, China). Female mice, 8 weeks old at the beginning of the BLM treatment, were used. All the experiments were conducted in accordance with the National Institutes of Health Guide for Care and Use of Laboratory Animals and were approved by the Bioethics Committee of the Shanghai Institute of Materia Medica **(**IACUC protocol # 2018-10-TW-12 for C57BL/6 mice).

### BLM-induced mice model of SSc

BLM (TargetMol, Boston, USA) was dissolved in phosphate buffer saline (PBS, Sigma-Aldrich, St. Louis, MO, USA) at the concentration of 300 μg/ml and sterilized by filtration. BLM (1.5 mg/kg) was injected subcutaneously into a single location on the shaved backs of C57BL/6 mice with an insulin needle. The injection was carried out consecutively for 4 weeks depending on the purpose of experiments.

### Experimental design

**Fig. 1:**
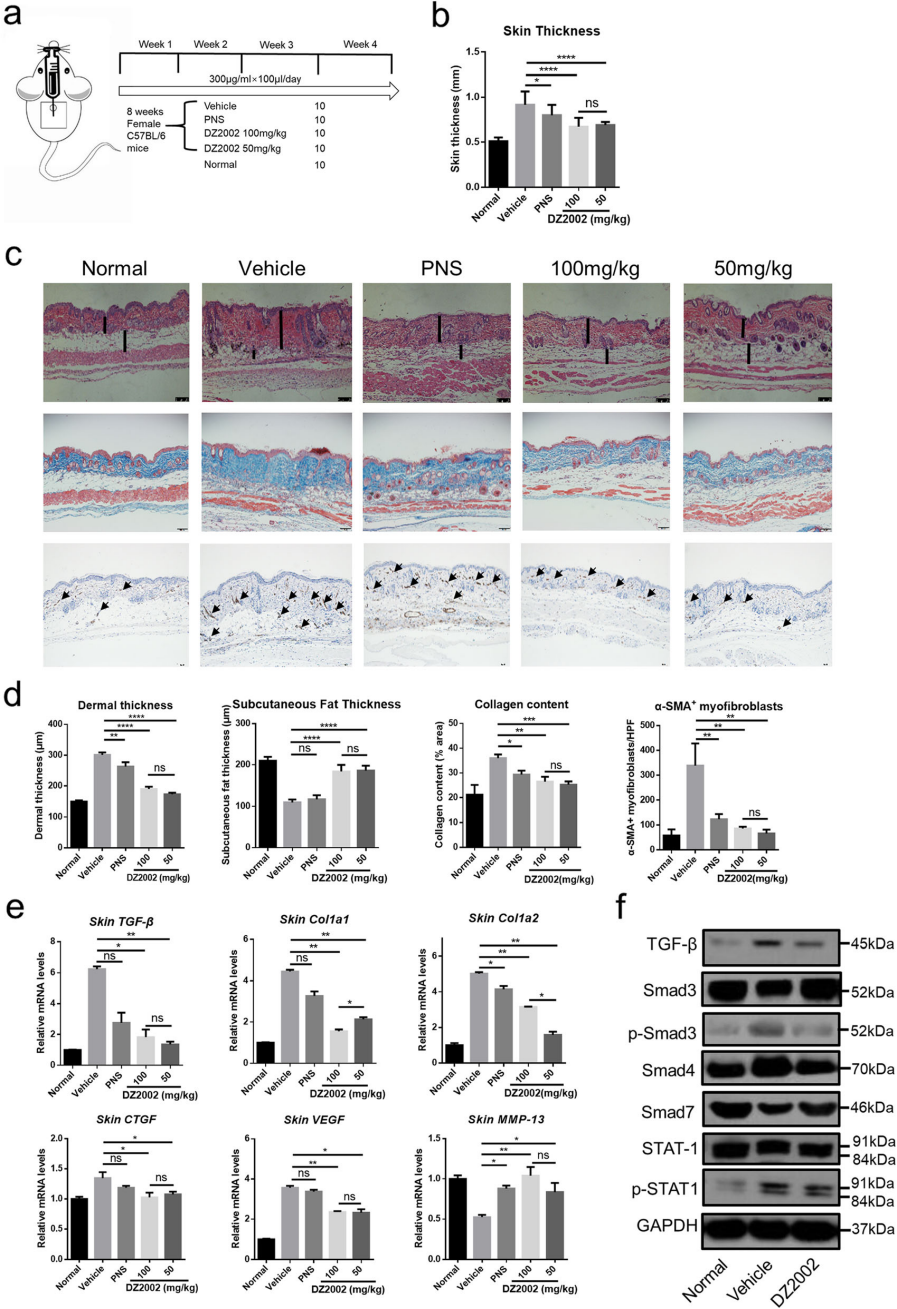
DZ2002 attenuated skin fibrosis and TGF-β signaling activation in a BLM-induced SSc mice model. **a** Experimental design of BLM-induced SSc mice model and drug treatment. **b** Total skin thickness of the back of each group of mice (*n* = 10 per group). **c** Representative skin sections stained with hematoxylin and eosin stain (top, original magnification, × 100), Masson’s trichrome stain (middle, original magnification, × 100), and α-SMA immunohistochemical staining (bottom, arrows represent α-SMA, original magnification, × 100) (scale bar = 100 μm). **d** Dermis thickness and subcutaneous fat layer thickness were measured on H&E stained images (*n* = 10 per group). **e** mRNA levels of molecules related to extracellular matrix metabolism, such as TGF-β, Col1a1, Col1a2, CTGF, VEGF, and MMP-13, in the lesional skin were assessed by quantitative real-time reverse transcription-PCR. **f** TGF-β, Smad3, p-Smad3, Smad4, Smad7, STAT1, and p-STAT1 proteins in the skin of C57BL/6 mice from the normal group, vehicle-treated group, and DZ2002-treated group (DZ2002: 50 mg/kg, 8 mice skin tissue mixed proteins). Mean ± SEM. HPF, high-power field, *P < 0.05, **P < 0.01, ***P < 0.001, ****P < 0.0001. PNS, prednisolone; d, day; ns, no significance

DZ2002 was synthesized at Shanghai Institute of Materia Medica (Shanghai, China). For therapy, 50 female C57BL/6 mice were randomly divided into five groups as follows: normal (*n* = 10), vehicle (ddH2O, *n* = 10), prednisolone (PNS) 2 mg/kg (*n* = 10), DZ2002 100 mg/kg (*n* = 10), and DZ2002 50 mg/kg (*n* = 10). The immunosuppressive drug PNS was used as a positive control to evaluate the pharmacodynamic effects of DZ2002. Mice were treated with a once-daily dosing regimen by oral intragastric administration from 8 weeks to 12 weeks of age. The dosage (milligrams per kilogram) listed is the dose per administration, and the doses were administered each day at 10:00 a.m. DZ2002 was dissolved and PNS was dispersed in ddH2O (Fig. 1a). At the end of the experiment, mice were euthanized to obtain skin for analysis.

### Histologic assessment and immunohistochemistry

All skin sections were taken from the paramidline, lower back region. Skin sections from BLM-induced SSc mice were taken together with hypodermal tissues. Sections were stained with hematoxylin and eosin and Masson’s trichrome stain. We examined dermal thickness, which was defined as the thickness of the skin from the top of the granular layer to the junction between the dermis and subcutaneous fat. Then we examined subcutaneous fat thickness. Immunohistochemistry was performed using antibodies directed against a-SMA (1:200, Cell Signaling Technology, Beverly, MA, USA), then with peroxidase-labeled secondary antibody (1:100, R&D systems, Minneapolis, MN, USA), followed by color development with the aminoethylcarbazole system. α-SMA (brown part) was counted under a high-power microscopic field. These pictures were analyzed by using Image-Pro Plus software (Media Cybernetics, Silver Springs, MD, USA). Each section was examined independently by two investigators in a blinded manner.

### Cell culture

Human dermal fibroblast cell line (BJ), human dermal microvascular endothelial cell line (HMEC-1) [31], and human acute monocytic leukemia cell line (THP-1) were purchased from ATCC (Manassas, VA, USA). BJ cells were cultured in DMEM (Gibco, Grand Island, NY, USA) containing 10% fetal bovine serum (FBS) (Gibco, Grand Island, NY, USA), 100 U/ml penicillin and 100 μg/ml streptomycin (Corning Incorporated, Corning, NY, USA) at 37 °C in a humidified 5% CO_2_ atmosphere. After adhering the cells, they were starved for 24 h with 0.1% FBS in DMEM, then stimulated with human TGF-β1 (PeproTech, Rocky Hill, NJ, USA) for 24 h. HMEC-1 cells were cultured in MCDB131 (without L-glutamine) as base medium (Sigma-Aldrich, St. Louis, MO, USA), supplemented with 10 ng/ml epidermal growth factor (Sigma-Aldrich, St. Louis, MO, USA), 1 μg/ml hydrocortisone (Sigma-Aldrich, St. Louis, MO, USA), 10 mM glutamine (Sigma-Aldrich, St. Louis, MO, USA),100 U/ml penicillin, 100 μg/ml streptomycin, and 10% FBS in 70 cm^2^ flasks until confluence at 37 °C, 5% CO_2_ atmosphere. After the cells were attached, TNF-α is stimulated for 24 h. THP-1 cells were cultured in RPMI 1640 (Gibco, Grand Island, NY, USA) containing 10% FBS, 100 U/ml penicillin, and 100 μg/ml streptomycin at 37 °C in a humidified 5% CO_2_ atmosphere.

### Cell Counting Kit-8 (CCK-8) cytotoxicity assay

Dispense 100 μl of cell suspension (BJ 2000 cells/well, HMEC-1 5000 cells/well) in a 96-well plate. Pre-incubate the plate for 24 h at 37 °C, 5% CO_2_ atmosphere. Add 100 μl of various concentrations of toxicant into the culture media in the plate. Incubate the plate for 24 h in the incubator. Add 20 μl of CCK-8 solution (Sigma-Aldrich, St Louis, MO, USA) to each well of the plate. Incubate the plate for 1–4 h in the incubator. Measure the absorbance at 450 nm using a microplate reader.

### BMDMs culture

Bone marrow-derived macrophages (BMDMs) were generated from bone marrow by macrophage colony-stimulating factor (M-CSF) induction, as described previously [32]. Bone marrow was aseptically flushed from the tibiae and femurs of euthanized mice and depleted of red blood cells using Red Blood Cell Lysis Buffer (Beyotime, Shanghai, China). Cells were incubated in IMDM (Gibco, Grand Island, NY, USA) in a cell culture dish at 37 °C for 2 h to remove macrophages. Nonadherent cells were resuspended in IMDM supplemented with 10% heat-inactivated FBS, 100 U/ml penicillin, 100 mg/ml streptomycin, 2 mM L-glutamine (Thermo Fisher Scientific, Pittsburgh, PA, USA), and 10 ng/ml M-CSF (PeproTech, Rocky Hill, NJ, USA) and cultured for 7 days. Nonadherent cells were removed, and M-CSF-conditioned medium was changed on day 3. To acquire M1 macrophages, macrophages were stimulated with 10 ng/ml IFN-γ (PeproTech, Rocky Hill, NJ, USA) and 1 μg/ml lipopolysaccharide (LPS, Sigma-Aldrich, St Louis, MO, USA) for 48 h for mRNA analysis or protein assays. To acquire M2 macrophages, macrophages were stimulated with 40 ng/ml IL-4 (PeproTech, Rocky Hill, NJ, USA) for 48 h for mRNA analysis or protein assays.

### Cell adhesion assay for HMEC-1 and THP-1

HMEC-1 (5 × 10^4^ cells/ml) were seeded in 24-well plates and were activated with TNF-a (40 ng/ml) in the presence or absence of DZ2002 for 6 h. THP-1 were labeled with 5 μM Calcein AM (BD Pharmingen, San Diego, CA, USA) in RPMI 1640 medium with 10% FBS for 30 min. The labeled THP-1 cells were washed with PBS three times, resuspended in endothelial cell medium and then added (1 × 10^5^ cells) onto HMEC-1. The co-culture system was incubated for 30 min at 37 °C in a humidified atmosphere of 5% CO_2_. The cells were washed with PBS three times to remove unadhered cells and were fixed with 3.7% formaldehyde for 15 min. The cells were visualized and imaged under an inverted fluorescent microscope (Olympus IX-73) and were counted in three microscopic fields of each well. These pictures were analyzed by using Image-Pro Plus software. Each section was examined independently by two investigators in a blinded manner.

### Preparation of skin cell suspension

A 2 × 2 cm^2^ piece of depilated back skin was minced and then digested in 6 ml of RPMI 1640 medium containing 10% FBS and 2 mg/ml crude collagenase (Sigma-Aldrich, St. Louis, MO, USA), 1.5 mg/ml hyaluronidase (Sigma-Aldrich, St. Louis, MO, USA), and 0.03 mg/ml DNase I (Roche Applied Science, Indianapolis, IN, USA) at 37 °C for 120 min [33]. Samples were passed through a 70-μm Falcon cell strainer (BD Biosciences, San Jose, CA, USA) to obtain single-cell suspensions. After centrifugation at 1200 rpm for 5 min, the cell pellet was resuspended in a RPMI 1640 medium. The cells were passed through a 40-μm Falcon cell strainer. The harvested cells were washed with ice-cold PBS and used for flow cytometric analysis and real-time PCR analysis.

### Western blot analysis

Primary skin cells were homogenized in sodium dodecyl sulfate sample buffer containing proteinase and phosphatase inhibitor. Stimulated BJ cells or HMEC-1 cells were lysed with RIPA lysis buffer (Beyotime, Shanghai, China). Homogenate were collected, and protein concentrations were then measured using the BCA assay kit (Thermo Fisher Scientific, Pittsburgh, PA, USA). The total protein samples were separated by sodium dodecyl sulfate-polyacrylamide gel electrophoresis and transferred to nitrocellulose membranes (Amersham Pharmacia Biotech, Buckinghamshire, UK). After blocking, the membranes were incubated with anti-TGF-β, anti-Smad3, anti-phospho-Smad3, anti-Smad4, anti-Smad7, anti-STAT1, anti-phospho-STAT1, anti-inducible NO synthase (iNOS), anti-Arginase 1 (Arg-1), anti-ICAM-1, and anti-VCAM-1 (all from Cell Signaling Technology, Beverly, MA, USA) (Mouse specific ICAM-1, Abcam, Cambridge, UK). After washing with TBS with Tween-20, the secondary antibodies (1:10000, Bio-Rad, Richmond, CA, USA) were added, and HRP-conjugated monoclonal mouse anti-GAPDH (1:10000, Kangcheng, Shanghai, China) was used as a control for normalization. Signals were detected with an ECL system (Amersham Bioscience, Buckinghamshire, UK) and exposed to classic autoradiography film.

### Cytokine analysis by ELISA

Skins from mice in each group were homogenized with lysis buffer to extract total protein. The concentration of total protein was determined by the BCA protein assay kit, collecting supernatant after BMDMs culture for 48 h. The levels of IL-1β, IL-4, IL-6, IL-10, IL-12p40, IL-17A, TNF-α, and IFN-γ in skin homogenates and cell culture supernatants were quantified by ELISA kits according to the manufacturer’s protocol (BD Pharmingen, San Diego, CA, USA).

### Real-time PCR

In order to obtain gene expression of certain proteins in tissues and cells, mRNA was isolated from the skin of tested mice with a Total RNA kit (Tiangen, Shanghai, China) and reverse transcribed with an RT Master Mix kit (Takara, Dalian, China). QRT-PCR was performed with the SYBR Premix Ex Taq kit (TOYOBO, Osaka, Japan) on an Applied Biosystems 7500 Fast Real-Time PCR System (Applied Biosystems, Foster city, CA, USA). Mouse β-actin and human GAPDH were used as a housekeeping gene. The sequences of primers were summarized in Additional file 1: Table S1.

### Flow cytometric analysis

Surface marker and intracellular transcription factor staining were conducted and analyzed using our previously reported methods [29]. Briefly, for surface marker and intracellular transcription factors, cells were collected and stained with FITC-, PE-, PerCP-Cy5.5-, APC-, BV421-, or BUV395-conjugated monoclonal antibodies (mAbs) for membrane molecules or intracellular staining after being blocked with anti-mouse CD16/CD32 (Fc Receptor Block, eBioscience, San Diego, CA, USA). For intracellular staining of cytokines, cells were cultured in the presence of GolgiStop (10 μg/ml, eBioscience, San Diego, CA, USA) for 6 h and then collected for the following staining. The immunofluorescent Abs used in this study were all from BD Biosciences (San Jose, CA, USA). Flow cytometric analysis was performed on a 2-laser/4-color FACS Calibur analytical cytometer or a 4-laser/13-color BD LSR II analytical cytometer (BD Biosciences, San Jose, CA, USA), and the data were analyzed with FlowJo software (Tree Star, Ashland, OR, USA).

### Immunofluorescence cytochemistry

BJ cells on coverslips were fixed in 4% paraformaldehyde (PFA) for 30 min and were permeabilized with 1% Triton X-100 for 10 min. After blocking with 3% BSA for 1 h, cells were incubated with rabbit anti-α-SMA (1:200) and anti-phosphorylated Smad3 (1:50; Santa Cruz Biotechnology, Santa Cruz, CA, USA) at 37 °C for 2 h. After washing with 1% PBS-Tween, Alexa Fluor 647-conjugated anti-rabbit secondary antibodies (BD Biosciences, San Jose, CA, USA) were added. Negative control reactions were included in each experiment and carried out by replacing primary antibodies with PBS. The cells were counterstained with 4′, 6-diamidino-2-phenylindole (DAPI, Abcam, Cambridge, UK). All images were captured using a Leica TCS SP8 STED confocal microscope. Skin tissues were embedded in OCT compound and sectioned on a cryostat (8 μm thick). After fixation in PFA, sections were blocked with 5% BSA for 60 min and then incubated with FITC-anti-CD3 (1:100, R&D systems, Minneapolis, MN, USA) or FITC-anti-CD11b (1:100, R&D systems, Minneapolis, MN, USA) at 4 °C overnight. The sections were counterstained with DAPI. Fluorescent cells were visualized and digital images were captured using a Leica TCS SP8 STED confocal microscope.

### Statistical analysis

One-way ANOVA followed by Dunnett’s multiple comparison test was used to compare parameters involving multiple groups, with GraphPad Prism 7.0 (GraphPad Software, San Diego, CA, USA) statistical software. *P* values less than 0.05 were considered significant.

## Results

### DZ2002 alleviates skin fibrosis in BLM-induced SSc mice model

We evaluated the effect of DZ2002 on BLM-induced dermal fibrosis. Compared to vehicle, DZ2002 significantly decreased skin thickness and dermal thickness in BLM-induced mice (Fig. 1b–d). As well, BLM-dependent decrease in subcutaneous fat thickness, which is usually seen in BLM-induced mice, was extraordinarily attenuated by DZ2002 administration (Fig. 1c, d). Consistent with this finding, DZ2002 treatment significantly reduced collagen accumulation and α-SMA expression in the dermis compared with the vehicle group (Fig. 1c, d). Meanwhile, DZ2002 treatment significantly reduced collagen content and mRNA expression of the Col1a1 and Col1a2 while promoting that of the matrix metalloproteinase-13 (MMP-13) in the lesional skin of BLM-induced mice (Fig. 1e). We also found that DZ2002 suppressed the mRNA expression of vascular endothelial growth factor (VEGF) in mice skin tissue (Fig. 1e). As hallmarks of SSc dermal fibroblasts elevated the expression, TGF-β and CTGF were further examined in experimental SSc mice [34, 35]. Remarkably, the decrease in mRNA expression of the TGF-β and CTGF was noted in BLM-induced mice exposed to DZ2002 (Fig. 1e), which was also confirmed at protein levels by western blot (Fig. 1f). Moreover, the downstream proteins of TGF-β such as p-Smad3, Smad4, and Smad7 also changed accordingly (Fig. 1f). We also evaluated the effects of DZ2002 on IFN-γ/STAT1 inflammatory activation signaling pathway. Notably, DZ2002 was able to strongly inhibit phosphorylation of STAT1 (Fig. 1f). These findings suggest that DZ2002 exerts a potent anti-fibrotic effect on dermal fibrosis by reducing the production of collagen, facilitating its degradation and regulating expression of various soluble factors in SSc mice model.

### DZ2002 lightens TGF-β-induced collagen accumulation in BJ cells

**Fig. 2:**
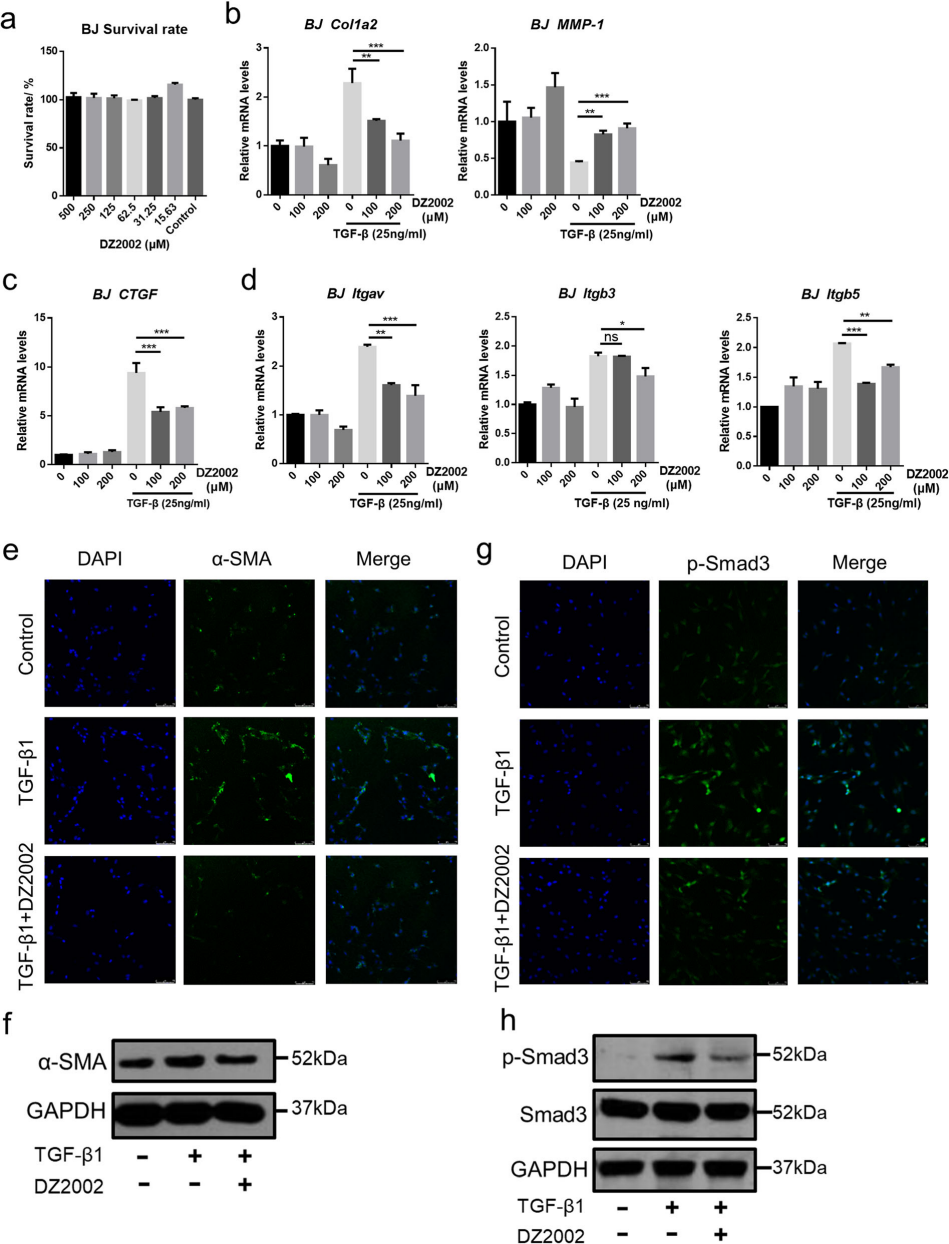
DZ2002 inhibited TGF-β dependent activation collagen accumulation of BJ cells. **a** Survival rate of BJ cells at different concentrations of DZ2002. **b**, **c** Col1a2, MMP-1, and CTGF mRNAs in the BJ cells were assessed by quantitative real-time reverse transcription-PCR. **d** Itgav, Itgb3, and Itgb5 mRNAs, encoding integrin αV, integrin β3, and integrin β5, were determined by quantitative real-time reverse transcription-PCR in BJ cells. **e** Immunofluorescence staining of α-SMA (green) in BJ cells (DAPI, blue, 24 h). **f** Western blot of α-SMA in BJ cells (24 h). **g** Immunofluorescence staining of p-Smad3 (green) in BJ cells (DAPI, blue, 2 h). **h** Western blot of p-Smad3 in BJ cells (2 h). **e**–**h** TGF-β = 25 ng/ml, DZ2002 = 200 μM. Mean ± SEM. *P < 0.05, **P < 0.01, ***P < 0.001. ns no significance

Here, we explored if DZ2002 directly affected the profibrotic phenotype of dermal fibroblasts in vitro. To evaluate the molecular mechanism, we stimulated BJ cells with recombinant active TGF-β1, which directly acts on TGF-β receptors. CCK-8 assay shows that DZ2002 at 500 μM and below had almost no toxicity to BJ cells (Fig. 2a). We mainly focused on the expression of type I collagen and matrix metalloproteinase-1 (MMP-1). DZ2002 significantly reversed the stimulatory effect of TGF-β on Col1a2 and MMP-1 mRNA levels dose-dependently in BJ cells (Fig. 2b). Additionally, DZ2002 also significantly reduced TGF-β1-induced mRNA expression of CTGF (Fig. 2c).

Since αVβ3 and αVβ5 integrins, latent receptor forms of TGF-β increase the sensitivity of dermal fibroblasts to TGF-β by recruiting and activating TGF-β on the cell surface [36–40]. Integrin αVβ3 and αVβ5 are upregulated by binding with latency-associated peptide on dermal fibroblasts of SSc patients [36]; therefore, we assessed the expression of these molecules in BJ cells. DZ2002 could be reduced to some extent the mRNA expression of integrin αV, β3, and β5 subunits (Fig. 2d), thus raising the possibility that DZ2002 may attenuate the establishment of TGF-β signaling in response to dermal fibroblasts. Additionally, under the same condition, DZ2002 reduced TGF-β1-induced expression of α-SMA protein (Fig. 2e, f). At the same time, the fibrin protein phosphorylated Smad3 of TGF-β downstream also had been inhibited (Fig. 2g, h). These results indicate that DZ2002 augments exogenous TGF-β-dependent CTGF induction through αVβ3 integrin- and αVβ5 integrin-dependent activation of TGF-β in dermal fibroblasts. Overall, these consequences reveal that DZ2002 inhibits fibrogenic genes, including Col1a2 and CTGF, at least partly through the integrin-mediated inhibition of TGF-β signal pathway.

### DZ2002 suppresses T cell activation and reduces inflammatory cell infiltration

**Fig. 3:**
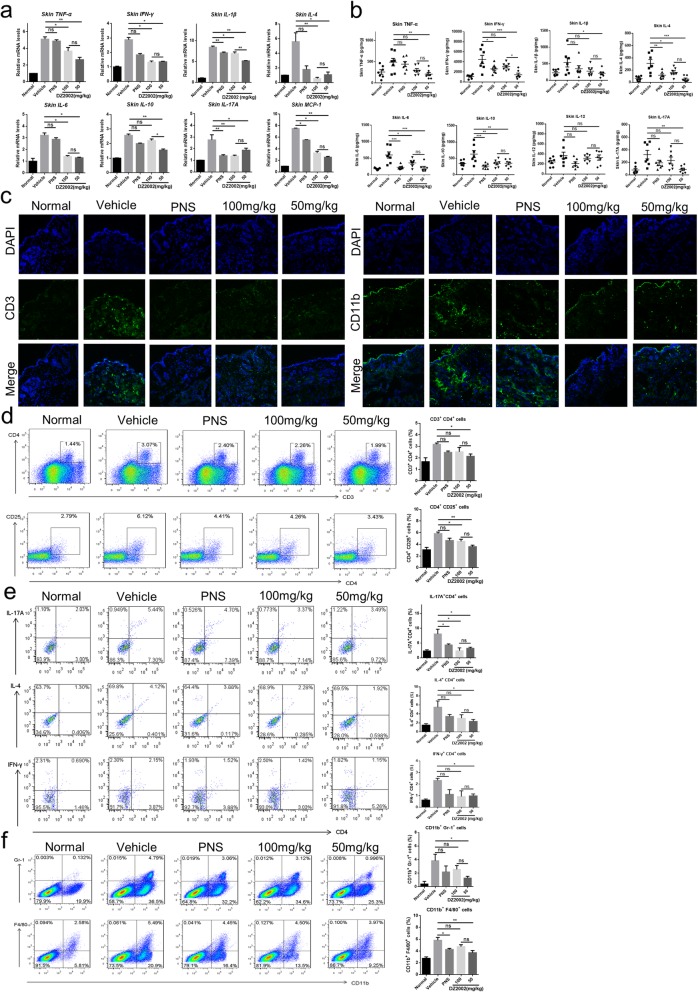
DZ2002 inhibited the activation of T cells and infiltration of neutrophils and macrophages in BLM-induced mice. **a** TNF-α, IFN-γ, IL-1β, IL-4, IL-6, IL-10, IL-17A, and MCP-1 mRNAs were determined by quantitative real-time reverse transcription-PCR in the BLM-induced SSc mice skin single-cell suspension. **b** Determination of TNF-α, IFN-γ, IL-1β, IL-4, IL-6, IL-10, IL-12, and IL-17A proteins in skin tissue homogenate supernatant of BLM-induced SSc mice by ELISA (*n* = 7 per group). **c** Immunofluorescence assay for CD3^+^ and CD11b^+^ cells in skin frozen sections of BLM-induced SSc mice. **d** Left: The percentages of CD3^+^ CD4^+^ cells and CD4^+^ CD25^+^ cells were measured by flow cytometry; Right: Percentage statistics of CD3^+^ CD4^+^ cells and CD4^+^ CD25^+^ cells (2 groups and *n* = 5 per group). **e** Left: Representative staining analysis of intracellular IL-17, IL-4, and IFN-γ in CD4^+^ T cells by flow cytometry; Right: Percentage statistics of IL-17A^+^ CD4^+^ cells, IL-4^+^ CD4^+^, and IFN-γ^+^ CD4^+^ cells (2 groups and *n* = 5 per group). **f** Left: The percentages of neutrophils (CD11b^+^ Gr-1^+^) and macrophages (CD11b^+^ F4/80^+^) were measured by flow cytometry; percentage statistics of CD11b^+^ Gr-1^+^ cells and CD11b^+^ F4/80^+^ cells (2 groups and *n* = 5 per group). Mean ± SEM, PNS prednisolone. *P < 0.05, **P < 0.01, ***P < 0.001, ns no significance

To investigate the impact of DZ2002 on BLM-induced inflammatory conditions, we next examined the expression of pro-inflammatory cytokines, T helper cytokines and chemokines, including TNF-a, IFN-γ, IL-1β, IL-4, IL-6, IL-10, IL-17A, and monocyte chemotactic protein 1 (MCP-1) in the lesional skin of BLM-induced mice. Notably, a significant reduction of mRNA expression due to DZ2002 treatment was observed in all the genes (Fig. 3a). To further elucidate the expression of these cytokines in skin tissue, we extracted the skin tissue homogenate. Consistently, DZ2002 inhibited the secretion of cytokines in the skin tissue (Fig. 3b). These indicated that DZ2002 widely suppressed the expression of pro-inflammatory, Th1, Th2, and Th17 cytokines, and chemokines in the context of BLM-induced mice skin. Immunofluorescence analysis demonstrated that the CD11b^+^ and CD3^+^ fluorescence intensity (green) in the skin were reduced after DZ2002 treatment compared with the vehicle control (Fig. 3c). To further assess if DZ2002 diversely affects the differentiation of T helper cells, we carried out flow cytometric analysis with skin cells. Of note, DZ2002 restored the BLM-dependent increase in the CD4^+^ T lymphocyte and CD25-activated T lymphocyte number in the skin (Fig. 3d). At the same time, DZ2002 inhibited the differentiation of Th1, Th2, and Th17 cells as well as the infiltration of neutrophils and macrophages (Fig. 3e, f). These data suggest that DZ2002 supports the inhibition of an SSc-like immune response in the lesional skin of BLM-induced mice.

### DZ2002 suppresses M1 and M2 polarization of macrophages

**Fig. 4:**
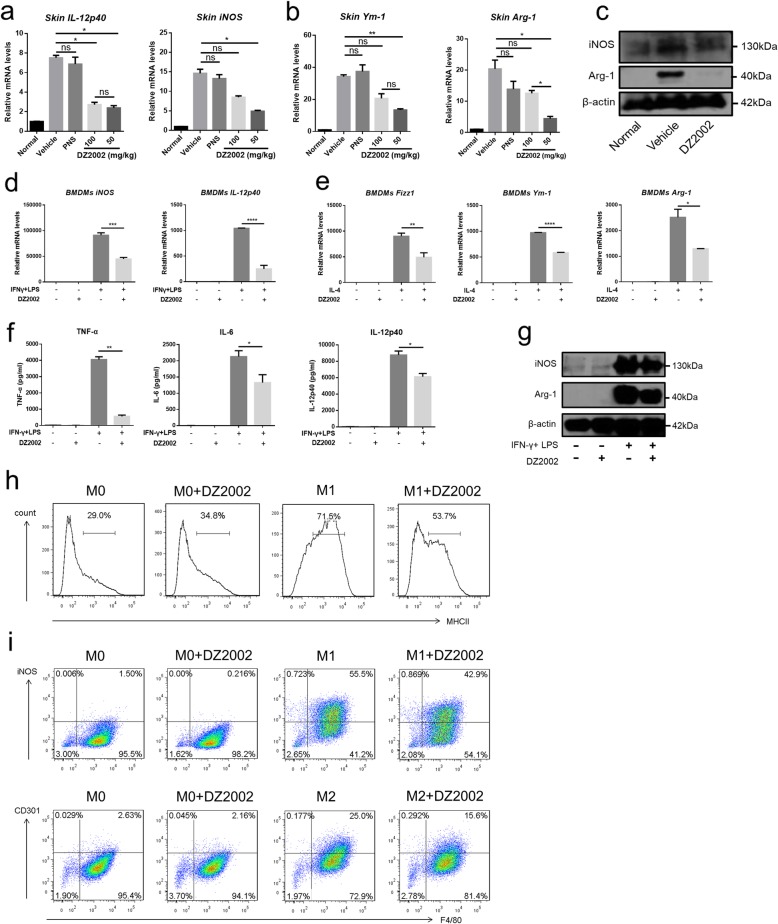
DZ2002 inhibited macrophage activation in vivo and in vitro. **a** IL-12p40 and iNOS mRNAs of M1 makers were determined by quantitative real-time reverse transcription-PCR of single-cell suspension in the BLM-induced SSc mice skin. **b** Ym-1 and Arg-1 mRNAs of M2 makers were determined by quantitative real-time reverse transcription-PCR of single-cell suspension in the BLM-induced SSc mice skin. **c** Western blot of iNOS and Arg-1 of single-cell suspension in the BLM-induced SSc mice skin (DZ2002 = 50 mg/kg). **d** IL-12p40 and iNOS mRNAs of M1 makers were determined by quantitative real-time reverse transcription-PCR in the BMDMs. **e** Fizz1, Ym-1, and Arg-1 mRNAs of M2 makers were determined by quantitative real-time reverse transcription-PCR in the BMDMs. **f** TNF-α, IL-6, and IL-12p40 cytokines in the supernatant secreted by BMDMs were determined by ELISA (*n* = 4 per group). **g** Western blot of iNOS and Arg-1 in the BMDMs (DZ2002 = 200 μM). **h** The percentages of MHCII of M1 were measured by flow cytometry. **i** The percentages of CD11b^+^ iNOS^+^ (M1 makers) and CD11b^+^ CD301^+^ (M2 makers) were measured in the BMDMs by flow cytometry. Mean ± SEM. *P < 0.05, **P < 0.01, ***P < 0.001; LPS lipopolysaccharide, PNS prednisolone, ns no significance

We evaluated the impact of DZ2002 on macrophages in the context of BLM-induced mice. DZ2002 significantly reduced mRNA expression of iNOS and IL-12p40; established polarization markers for M1 macrophages, also to chitinase 3-like 3 (Ym-1) and Arg-1; and established polarization markers for M2 macrophages, in the skin lesions of BLM-induced mice (Fig. 4a, b). The expression of iNOS and Arg-1 proteins also supported the findings (Fig. 4c). We used normal mouse BMDMs to examine the effect of DZ2002 on macrophage differentiation in vitro. DZ2002 also significantly reversed the IFN-γ and lipopolysaccharides-trigged iNOS and IL-12p40 mRNA expression and IL-4-induced Fizz1, Ym-1, and Arg-1 mRNA expression (Fig. 4d, e). iNOS and Arg-1 proteins are also inhibited by DZ2002 (Fig. 4g). And in the supernatant of BMDMs, DZ2002 also significantly inhibited TNF-α, IL-6, and IL-12p40 (M1 makers) production (Fig. 4f). Similarly, we also used flow cytometry to verify that DZ2002 reduced the proportion of MHCII (Fig. 4h). And the proportion of F4/80^+^ iNOS^+^ (M1 makers) and F4/80^+^ CD301^+^ (M2 makers) cells was significantly reduced (Fig. 4i). Taken together, these results indicate that DZ2002 inhibits the differentiation of M1 macrophages and M2 macrophages in vivo and in vitro to contribute to anti-inflammatory and anti-fibrosis.

### DZ2002 reduces adhesion molecule expression in vivo and in vitro

**Fig. 5:**
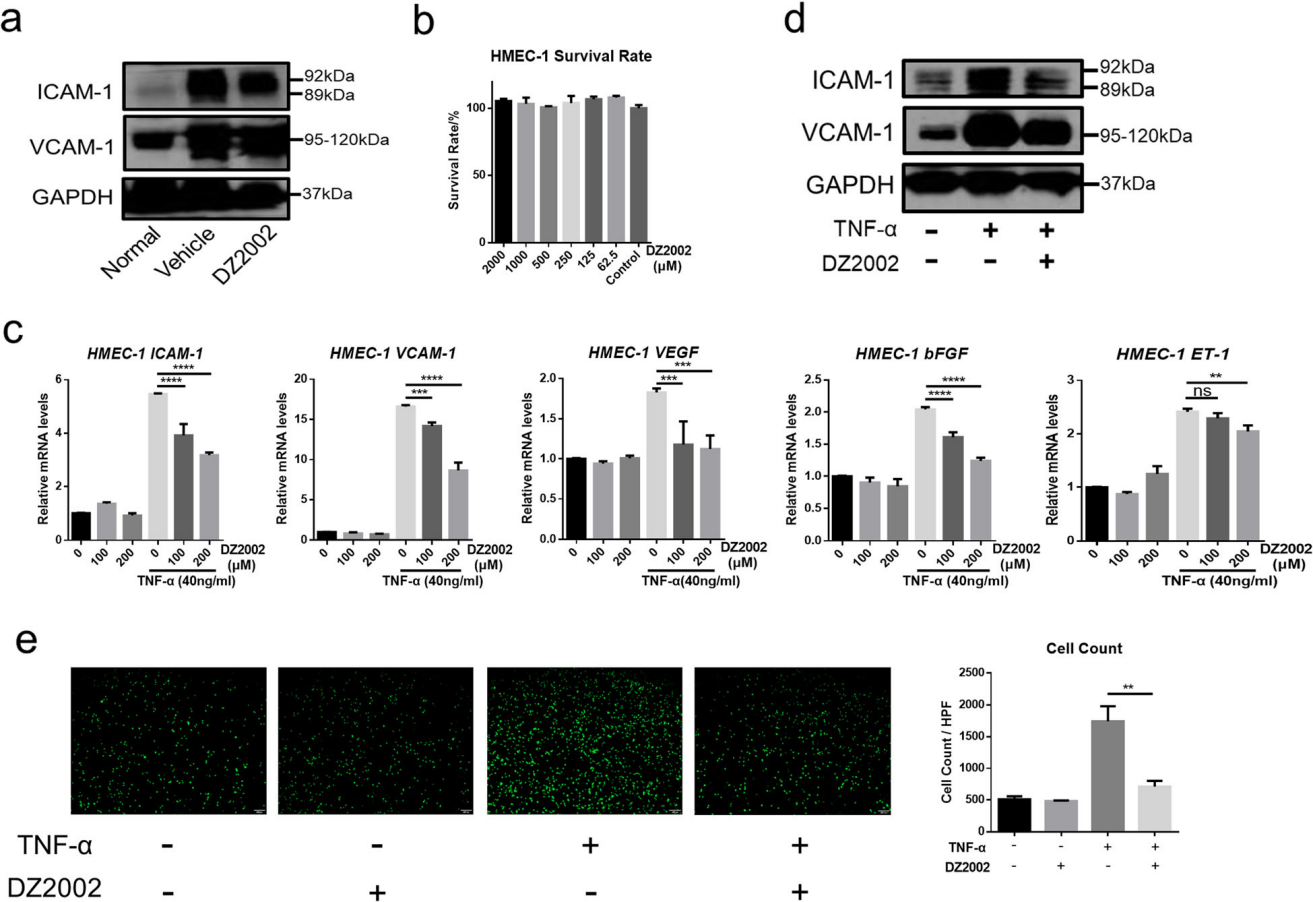
DZ2002 reduced ICAM-1 and VCAM-1 in vivo and in vitro. **a** Western blot of ICAM-1 and VCAM-1 in BLM-induced SSc mice skin (DZ2002 = 50 mg/kg). **b** Survival rate of HMEC-1 at different concentrations of DZ2002 treatment. **c** Evaluation of mRNA levels of molecules associated with the upregulation of endothelial adhesion molecules in HMEC-1 by quantitative real-time reverse transcription-PCR, such as ICAM-1, VCAM-1, VEGF, bFGF, and ET-1. **d** Western blot of ICAM-1 and VCAM-1 in the HMEC-1 cells (TNF-α = 40 ng/ml, DZ2002 = 200 μM). **e** Representative photomicrographs showing the stained THP-1 cells on HMEC-1 cell layer (× 40, scale bar = 100 μm). The HMEC-1 cells were stimulated with TNF-a (40 ng/ml) with or without DZ2002 (200 μM). After 6 h incubation, those stained THP-1 cells adhered on HMEC-1 cell layer were counted. Mean ± SEM. *P < 0.05, **P < 0.01, ***P < 0.001, ****P < 0.0001; ns no significance

We examined the effect of DZ2002 on the expression of ICAM-1 and VCAM-1 in BLM-induced mice. As expected, ICAM-1 and VCAM-1 protein expression in the skin lesions of BLM-induced mice was significantly reduced by DZ2002 treatment (Fig. 5a). In vitro, we used TNFα-stimulated HMEC-1 to study the effects of DZ2002 on vascular injury. CCK-8 assay shows that DZ2002 at 2000 μM and below had almost no toxicity in HMEC-1 cells (Fig. 5b). Notably, DZ2002 obviously reduced the adhesion molecule markers ICAM-1, VCAM-1, VEGF, basic fibroblast growth factor (bFGF), and ET-1 mRNA expression in a dose-dependent manner (Fig. 5c). And ICAM-1 and VCAM-1 proteins were also alleviated in HMEC-1 (Fig. 5d).

Vascular endothelial cells recruit circulating monocytes to regions of vascular injury and/or infection [41]. Following their entry into the vessel wall, monocytes differentiate into macrophages, which drive an inflammatory response to neutralize invading pathogens, repair tissue damage or activate other immune cells [42]. Here, we used THP-1 and HMEC-1 to co-culture in vitro to simulate this environment. It could be seen that THP-1 adhered to HMEC-1 damaged after TNF-α stimulation and DZ2002 reversed THP-1 adhesion (Fig. 5e). These results indicated that DZ2002 repaired vascular endothelial injury and reduced endothelial cell adhesion molecule expression.

## Discussion

**Fig. 6:**
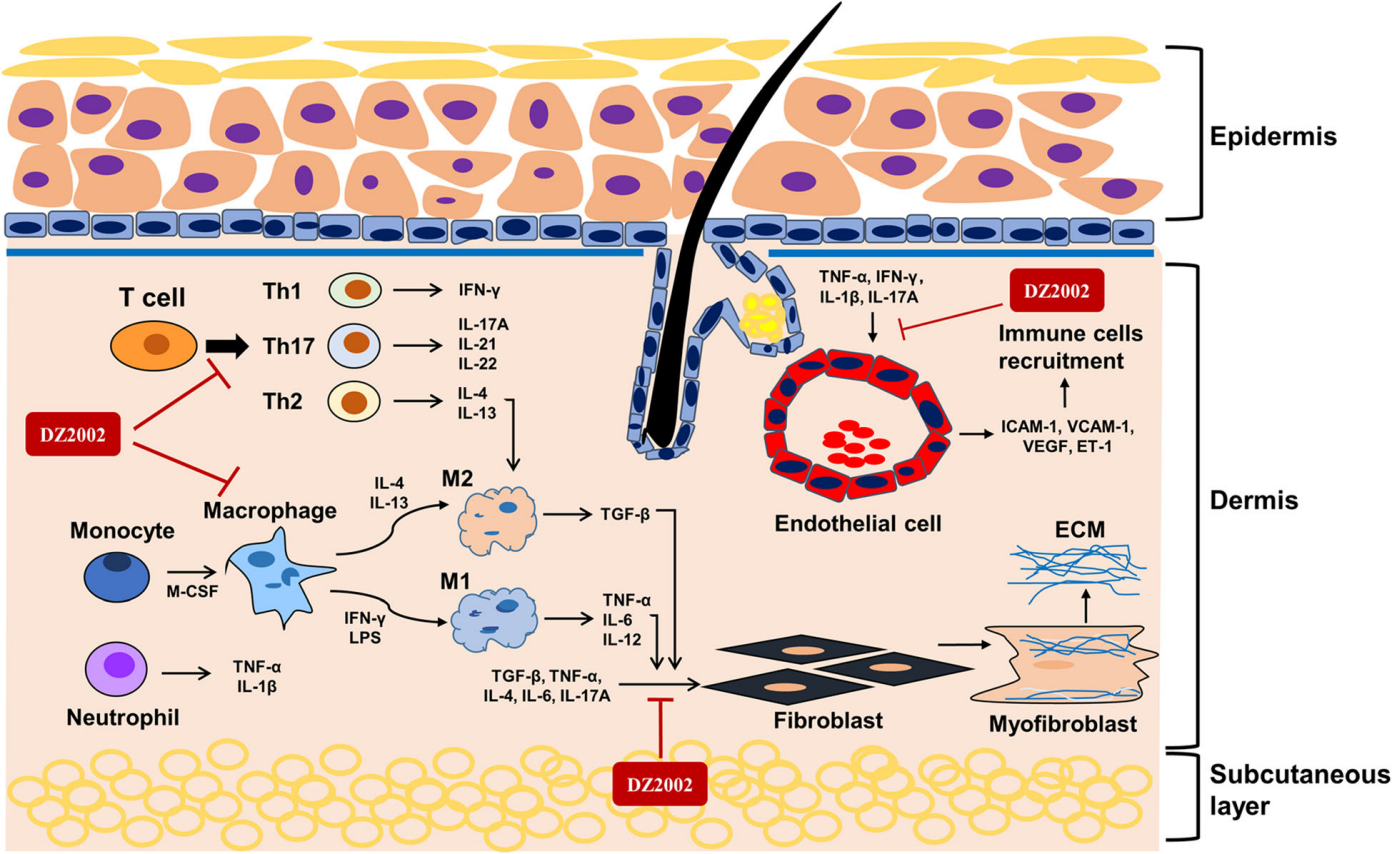
The pharmacodynamic mechanism of DZ2002 on experimental systemic sclerosis models

SSc is a chronic autoimmune disease that is characterized by diffuse fibrosis in the skin and internal organs. It is widely accepted that excessive deposition of ECM and vascular endothelial cell activation in SSc are highly associated with the inflammatory abnormalities [43]. Our previous research showed that DZ2002 had therapeutic effects on a variety of autoimmune diseases [25–28]. We hypothesized that its immunosuppressive activity might have a therapeutic effect in SSc. As hypothesized, DZ2002 inhibited the development of dermal fibrosis in BLM-induced mice. DZ2002 prevented BLM-induced dermal fibrosis by affecting pathological processes that broadly influenced the basis of tissue fibrosis, such as fibroblast activation, activation of TGF-β/Smad signaling pathway, Th1/Th2/Th17-skewed immune polarization, and M1 and M2 macrophages activation. In addition, DZ2002 inhibited the upregulation of microvascular endothelial cell adhesion molecules. These results signify that DZ2002 has the potential to modulate different SSc-related pathological features (Fig. 6).

TGF-β has been implicated to play a critical role in initiating and sustaining the fibrotic evolution in SSc, a function mediated through both Smad-dependent and Smad-independent pathways [3]. Fibroblasts of SSc patients show constitutive Smad2/3 phosphorylation and nuclear localization, and various anomalous Smad signals are over activated. Thus, targeting TGF-β/Smad signal pathway is an effective strategy for the treatment of SSc. Our data showed that DZ2002 inhibited the phosphorylation of Smad3 protein in BLM-injected SSc mice skin and human dermal fibroblasts. The inhibition of Smad3 phosphorylation, thus interfering with its binding to Smad-binding elements, might lead to reduced transcription of fibrogenic genes such as Col1a1, Col1a2, and CTGF, as seen in human fibroblasts in vitro. Additionally, our limited data suggested that DZ2002 suppressed STAT1 phosphorylation in BLM-injected mice skin.

BLM-injection characteristically induces Th2/Th17-skewed immune polarization in fibrotic tissues, leading to fibroblasts activation [44]; nevertheless, DZ2002 treatment diversely reduced mRNA and protein levels of cytokines produced by Th1, Th2, and Th17 cells in the lesional skin of BLM-induced mice. Under the same condition, the proportion of Th1, Th2, Th17, and activated T cells was decreased, suggesting that DZ2002 attenuated the inflammatory state in response to BLM-induced tissue injury by inhibiting the differentiation of T cells such as Th1, Th2, and Th17 cells. Our previous study also showed that DZ2002 prevented T cell differentiation by affecting the function of bone marrow-derived dendritic cells (BMDCs) in BMDC-T cell co-culture systems [45]. In addition to the altered T cell subsets, the proportion of inflammatory cells, including neutrophils and macrophages, was attenuated in response to DZ2002 in the lesional skin of BLM-induced mice, which was probably to be at least partially credited to the DZ2002-dependent inhibition of MCP-1. DZ2002 also inhibited macrophages differentiating to a pro-inflammatory M1-like phenotype and a profibrotic M2-like phenotype in the lesional skin of BLM-induced mice partly by directly acting on macrophages, and these were proved by in vitro experiments on BMDMs. DZ2002 significantly inhibited M1 macrophage markers (IL-12p40 and iNOS) and M2 macrophage markers (Fizz1, Ym-1, Arg-1) in BMDMs, also proved by flow cytometry. Considering the effects of DZ2002 on the macrophage phenotype in BLM-treated mice and BMDMs, we draw a conclusion that DZ2002 might exert anti-fibrotic effects on pathological fibrosis partly by inhibiting macrophage differentiation into M1 and M2 phenotypes. Also, DZ2002 ameliorated the upregulation of human microvascular endothelial cell adhesion molecules, including ICAM-1, VCAM-1, and VEGF. This was partly due to the inhibition of inflammatory cytokines by DZ2002, which reduced the recruitment of monocytes to repair vascular damage. Moreover, we confirmed the direct anti-fibrotic effect of DZ2002 on dermal fibroblasts stimulated with TGF-β1.

To summarize, this study confirmed the exclusive anti-fibrotic effects of DZ2002 in the context of BLM-induced dermal fibrosis. Of note, the multi-faceted inhibition effects of this agent on the TGF-β-induced human dermal fibroblasts fibrosis process have not been reported previously. Also, DZ2002 improved vasculopathy through inhibiting adhesion molecule expression. These data in vivo and in vitro demonstrate that DZ2002 exerts an effective therapeutic effect on the experimental SSc models by preventing the three main components of the pathogenesis of SSc.

## Conclusions

The present study demonstrates that DZ2002 has anti-inflammatory, anti-fibrotic, and anti-angiogenic effects on experimental SSc models by acting on BLM-induced mice and various types of cells, including fibroblasts, endothelial cells, and immune cells. These indicate DZ2002 is expected to become a potential new drug for the treatment of SSc.

## Supplementary information

**Additional file 1: Table S1.** Specific primers used in real-time PCR analysis.

## Data Availability

Not applicable

## Abbreviations

SAHH: S-adenosyl-l-homocysteine hydrolase
BLM: Bleomycin
SSc: Systemic sclerosis
TGF-β: Transforming growth factor-β
IL: Interleukin
CTGF: Connective tissue growth factor
ET-1: Endothelin-1
ECM: Extracellular matrix
α-SMA: α-Smooth muscle actin
Col1a1: Collagen type I α 1
TNF-α: Tumor necrosis factor-α
IFN-γ: Interferon-γ
Th: T helper
ICAM-1: Intercellular adhesion molecule-1
VCAM-1: Vascular cell adhesion molecule 1
SAH: S-adenosyl-l-homocysteine
SAM: S-adenosylmethionine
PNS: Prednisolone
BJ: Human dermal fibroblast cell line
HMEC-1: Human dermal microvascular endothelial cell line
THP-1: Human acute monocytic leukemia cell line
FBS: Fetal bovine serum
CCK-8: Cell Counting Kit-8
BMDMs: Bone marrow-derived macrophages
M-CSF: Macrophage colony-stimulating factor
LPS: Lipopolysaccharide
Arg-1: Arginase 1
Ym-1: Chitinase 3-like 3
iNOS: Inducible NO synthase
PFA: Paraformaldehyde
DAPI: 4′,6-Diamidino-2-phenylindole
MMP: Matrix metalloproteinase
MCP-1: Monocyte chemotactic protein 1
bFGF: Basic fibroblast growth factor
Fizz1: Found in inflammatory zone 1
HPF: High-power field
