# Supplementary material for: DZ2002 ameliorates fibrosis, inflammation, and vasculopathy in experimental systemic sclerosis models
Source: Arthritis Res Ther. 2019 Dec 16;21:290. doi: 10.1186/s13075-019-2074-9 (PMC6916442; doi:10.1186/s13075-019-2074-9)
Supplement: Supplementary file 1 — Additional file 1: Table S1. Specific primers used in real-time PCR analysis. [file 13075_2019_2074_MOESM1_ESM.docx]

| **Table S1. Specific primers used in real-time PCR analysis** | | |
| --- | --- | --- |
| Gene | Primer | Sequence (5'-3') |
| m β-actin | Forward | GGCTGTATTCCCCTCCATCG |
|  | Reverse | CCAGTTGGTAACAATGCCATGT |
| m TGF-β1 | Forward | GCAACATGTGGAACTCTACCAGAA |
|  | Reverse | GACGTCAAAAGACAGCCACTCA |
| m Col1a1 | Forward | GCCAAGAAGACATCCCTGAAG |
|  | Reverse | TGTGGCAGATACAGATCAAGC |
| m Col1a2 | Forward | GCAGGTTCACCTACTCTGTCCT |
|  | Forward | CTTGCCCCATTCATTTGTCT |
| m VEGF | Reverse | TATTCAGCGGACTCACCAGC |
|  | Forward | AACCAACCTCCTCAAACCGT |
| m MMP-13 | Reverse | GCCAGAACTTCCCAACCAT |
|  | Forward | TCAGAGCCCAGAATTTTCTCC |
| m IL-1β | Reverse | GCAACTGTTCCTGAACTCAACT |
|  | Forward | ATCTTTTGGGGTCCGTCAACT |
| m IL-4 | Forward | CAACGAAGAACACCACAGAG |
|  | Reverse | GGACTTGGACTCATTCATGG |
| m IL-6 | Forward | CTGCAAGAGACTTCCATCCAG |
|  | Reverse | AGTGGTATAGACAGGTCTGTTGG |
| m IL-10 | Forward | TTTGAATTCCCTGGGTGAGAA |
|  | Reverse | ACAGGGGAGAAATCGATGACA |
| m IL-12p40 | Forward | TGGTTTGCCATCGTTTTGCTG |
|  | Reverse | ACAGGTGAGGTTCACTGTTTCT |
| m IL-17A | Forward | CTCCAGAAGGCCCTCAGACTAC |
|  | Reverse | AGCTTTCCCTCCGCATTGACACAG |
| m TNF-α | Forward | CCCTCACACTCAGATCATCTTCT |
|  | Forward | GCTACGACGTGGGCTACAG |
| m IFN-γ | Reverse | TGCTGATGGCCTGATTGTCTT |
|  | Forward | GCCACGGCACAGTCATTGA |
| m MCP-1 | Reverse | CATCCACGTGTTGGCTCA |
|  | Forward | GATCATCTTGCTGGTGAATGAGT |
| m CTGF | Reverse | GTGCCAGAACGCACACTG |
|  | Forward | CCCCGGTTACACTCCAAA |
| m Arg-1 | Reverse | CAGAAGAATGGAAGAGTCAG |
|  | Forward | CAGATATGCAGGGAGTCACC |
| m iNOS | Reverse | GTTCTCAGCCCAACAATACAAGA |
|  | Forward | GTGGACGGGTCGATGTCAC |
| m Ym-1 | Forward | GGGCATACCTTTATCCTGAG |
|  | Reverse | CCACTGAAGTCATCCATGTC |
| m Fizz1 | Forward | TCCCAGTGAATACTGATGAGA |
|  | Reverse | CCACTCTGGATCTCCCAAGA |

| Gene | Primer | Sequence (5'-3') |
| --- | --- | --- |
| h Col1a2 | Forward | GATGTTGAACTTGTTGCTGAGG |
|  | Reverse | TCTTTCCCCATTCATTTGTCTT |
| h MMP-1 | Forward | GCTAACCTTTGATGCTATAACTACGA |
|  | Reverse | GGATTTGTGCGCATGTAGAA |
| h CTGF | Forward | TTGCGAAGCTGACCTGGAAGAGAA |
|  | Reverse | AGCTCGGTATGTCTTCATGCTGGT |
| h Itgav | Forward | GCCGTGGATTTCTTCGTG |
|  | Reverse | GAGGACCTGCCCTCCTTC |
| h Itgb3 | Forward | CGCTAAATTTGAGGAAGAACG |
|  | Reverse | GAAGGTAGACGTGGCCTCTTT |
| h Itgb5 | Forward | GGAGTTTGCAAAGTTTCAGAGC |
|  | Reverse | TGTGCGTGGAGATAGGCTTT |
| h ICAM-1 | Forward | TAGAGACCCCGTTGCCTAAA |
|  | Reverse | TCATACACCTTCCGGTTGTTC |
| h VCAM-1 | Forward | GGAAGTGGAATTAATTATCCAA |
|  | Reverse | CTACACTTTTGATTTCTGTG |
| h ET-1 | Forward | GACATCATTTGGGTCAACACTC |
|  | Reverse | GGCATCTATTTTCACGGTCTGT |
| h bFGF | Forward | ATGGCAGCCGGGAGCATCACC |
|  | Reverse | CACACACTCCTTTGATAGACACA |
| h GAPDH | Forward | ACCCACTCCTCCACCTTTGA |
|  | Reverse | CATACCAGGAAATGAGCTTGACAA |

Remarks: m, mouse; h, human.
